# Synthesis, Characterization, and Preliminary Analysis of Squid Pen Trypsin Hydrolysates and Chitosan Microcapsules

**DOI:** 10.3390/ijms26072885

**Published:** 2025-03-22

**Authors:** Ruimin Li, Wenkui Song, Shijia Huang, Chuyi Liu, Mingbo Li, Leilei Sun

**Affiliations:** 1 Yantai Key Laboratory of Characteristic Agricultural Bioresource Conservation & Germplasm Innovative Utilization, School of Life Sciences, Yantai University, Yantai 264005, China; 17861135192@163.com (R.L.); sjhuang01@163.com (S.H.); limingbo1711@163.com (M.L.); 2 Guangdong Provincial Key Laboratory of Aquatic Products Processing and Safety, National Research and Development Branch Center for Shellfish Processing (Zhanjiang), Guangdong Provincial Engineering Technology Research Center of Seafood, Guangdong Province Engineering Laboratory for Marine Biological Products, College of Food Science and Technology, Guangdong Ocean University, Zhanjiang 524088, China; songwk@gdou.edu.cn; 3 Marine Biomedical Research Institute of Qingdao, Qingdao 266073, China; liucy@ouc.edu.cn; 4 College of Food Science and Engineering, Ocean University of China, Qingdao 266003, China

**Keywords:** microcapsules, squid pen trypsin hydrolysates, β-chitosan, structural characterization, antioxidant activity

## Abstract

Squid pen (SP) was found to contain 64.41% protein and 26.03% chitin. The amino acid composition revealed that Met was the most abundant amino acid in SP, with a concentration of 13.67 g/100 g. To enhance the stability and bioavailability of SP hydrolysates, microcapsules were developed using ultrasonic emulsification techniques with SP trypsin hydrolysates (SPTH) and SP β-chitosan (SPC). The optimal preparation conditions involved using a 2% concentration of SPC, a 4 mg/mL concentration of SPTH, a core-to-wall ratio (*v*/*v*) of 1:3 for SPTH/SPC, and subjecting them to ultrasonic treatment for 20 min. These microcapsules had a loading capacity of 58.95% for SPTH under these conditions. The successful encapsulation of SPTH in the SPC complex to form SPC-SPTH microcapsules was confirmed by FTIR, XRD, DSC, and SEM, exhibiting good thermal stability, small particle size, and high encapsulation efficiency. In vitro digestion studies demonstrated a release of 15.61% in simulated gastric fluid and 69.32% in intestinal fluid, achieving targeted release in the intestines. The digested products exhibited superior antioxidant activity compared to free SPTH digests, suggesting that microencapsulation effectively preserves SPTH bioactivity. This study enhances the bioavailability of SPTH and offers a promising delivery system for natural compounds with low bioavailability and stability.

## 1. Introduction

It has been reported that squid pen (SP) contains 30–50% β-chitin, 50–70% protein, and less than 1% ash [1]. Therefore, SP can be considered a valuable source for the preparation of protein hydrolysates and β-chitosan.

Bioactive substances like peptides or protein hydrolysates require protection from environmental factors such as pH, light, oxygen, temperature, and enzymatic degradation due to their chemical or physical instability [2]. Moreover, these compounds may degrade in the gastrointestinal system and not be effectively absorbed by the body [3]. In our previous study, SPTH, a protein, hydrolysates with high antioxidant activity, experienced a significant reduction in activity after in vitro simulated digestion [4]. To enhance the bioavailability of SPTH and achieve targeted release in intestinal fluids, microcapsule technology can be utilized [5].

Microencapsulation is a process utilized in the food industry to safeguard bioactive substances by encapsulating them within microscopic capsules for targeted release. The selection of capsule wall materials is crucial in determining the functionality of microcapsules. Biobased polymers, particularly chitosan, a cationic natural polysaccharide, have garnered attention due to the rise of natural materials and green chemistry. Chitosan is being explored as a promising material for capsule walls in delivering nutraceuticals owing to its natural, non-toxic, readily available, and renewable characteristics [6,7].

The most common method for extracting chitosan involves the deacetylation of chitin, which not only enhances its antibacterial activity but also improves its solubility in acidic environments [8]. Chitin typically exists in fibrous crystalline states with different polymorphs, including α, β, and γ forms [9]. β-chitin, predominantly found in SP, has polymer chains arranged in a parallel manner with weaker intermolecular hydrogen bonding, making it easier to deacetylate during chitosan preparation [10].

Chitosan-based microcapsules have garnered significant attention from researchers for their potential applications in drug delivery, sustained release, storage, and transportation. Dong et al. [11] conducted a study on preparing rutin microcapsules using soybean protein isolate and chitosan hydrochloride through composite coacervation. The study demonstrated improved stability and water solubility of rutin, enhancing its bioavailability. Pérez-Córdoba et al. [12] focused on gelatin-chitosan-based films with nanoemulsion-loaded active compounds, showing effectiveness against Pseudomonas aeruginosa and high antioxidant activity. Cheng et al. [13] utilized chitosan for microcapsules delivering celecoxib, enhancing drug absorption efficiency in animal experiments. Jiang et al. [14] developed pectin-chitosan collagen composite microcapsules with superior anti-swelling, anti-shrinkage, and sustained release capabilities.

There have been numerous studies and applications focused on chitosan derived from shrimp and crab shells. However, fewer applications have explored β-chitosan obtained from SP. Therefore, this research aimed to utilize β-chitosan derived from SP to create microcapsules containing SPTH and characterized these formulations in terms of their physical properties, spectroscopic features, microstructure, release properties, thermal properties, as well as their antioxidant activities. This study aligns with the sustainable utilization of biomass, which is both cost- and energy-effective. Furthermore, it provides an avenue for considering the future development and application of composite systems using β-chitosan derived from SP as wall material for microcapsules.

## 2. Results and Discussion

### 2.1. SP Composition

Table 1 illustrated that the Peruvian SP contained 6.02 ± 0.76% water, 3.26 ± 0.22% (dry basis) ash, 64.41 ± 1.24% protein, and 26.03 ± 0.73% chitin. Cortizo [15] also found similar results with Illex argentinus SP, showing 64% protein and 31% chitin.

Table 2 illustrated the amino acid composition of SP, revealing the presence of 7 essential amino acids and 10 non-essential amino acids, totaling 17 amino acids. Among the measured amino acids, Met had the highest content (13.67 g/100 g), followed by Tyr (9.81 g/100 g), His (7.12 g/100 g), and Pro (6.68 g/100 g). Met can synthesize Cys, ensuring metabolic steady with a sufficient dietary intake of Met [16]. The EAA/TAA ratio was 37.55%, and the EAA/NEAA ratio was 60.13%. These values align well with the FAO/WHO recommendation that high-quality protein should have an EAA/TAA ratio of around 40% and an EAA/NEAA ratio above 60% [17]. The amino acid composition of SP in this study appears to be well-balanced, indicating its potential as a valuable protein source.

### 2.2. Preparation Conditions and Encapsulation Efficiency of Microcapsules

The optimal processing conditions for preparing the microcapsules were determined through orthogonal testing. The optimal preparation conditions for SPTH-SPC microcapsules and their encapsulation efficiency are presented in Table 3. The SPC concentration was set at 2%, the concentration of SPTH at 4 mg/mL, the core-to-wall ratio at 1:3, and the sonication time at 20 min. Under these conditions, the encapsulation efficiency of the microcapsules was measured to be 58.95 ± 2.53%.

### 2.3. Bulk Density of Microcapsules

In relation to the angle of repose and fluidity, a range of 30° to 45° typically signifies improved product fluidity [18]. With the angle of repose of SPTH-SPC microcapsules measured at 30.1° ± 0.27° and a bulk density of 0.82 ± 0.01 g/cm^3^, it suggests that the prepared microcapsules exhibit enhanced fluidity. Additionally, the smooth surface of the powders and low viscosity further support this observation.

### 2.4. In Vitro Release Profiles During Simulated Gastrointestinal Digestion

**Figure 1:**
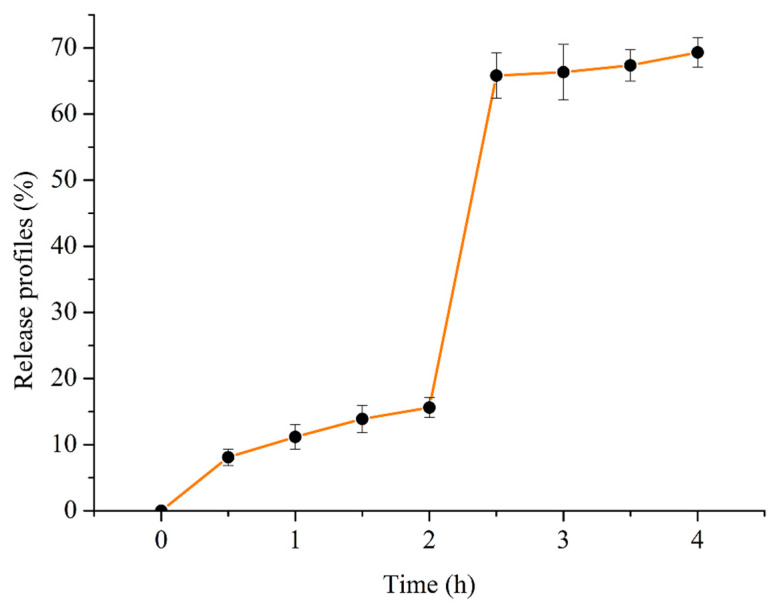
Release profiles of SPTH-SPC microcapsules.

The release profiles of SPTH-SPC microcapsules in simulated gastro-intestinal fluids were displayed in Figure 1. The percentage of SPTH released from SPTH-SPC microcapsules was 15.61% in the artificial gastric environment and 69.32% in the intestinal environment. The amount of free SPTH significantly increased (*p* < 0.05) after simulating intestinal digestion, suggesting that the SPC shell membrane of the microcapsules remained intact in the stomach environment before releasing SPTH. The finding was consistent with Zhao et al. [19]. Overall, SPTH-SPC microcapsules effectively encapsulated SPTH to prevent premature release, allowing more SPTH to reach the intestine. Fortunately, almost all contents were released after 4 h of gastrointestinal digestion. This study confirms the potential of SPTH-SPC microcapsules as a delivery system for small peptides, enabling encapsulation and controlled release of peptides. In β-chitosan, the protonation of amino groups, the rigidity of molecular chains, and the crystallinity collectively contribute to the slow swelling of chitosan-based wall materials in the gastric acid environment. This slow swelling results in relatively minor alterations in pore size and permeability of the wall materials, creating a significant impediment to the diffusion of SPTH and leading to a comparatively sluggish release rate. Consequently, SPTH maintains stability within the gastric acid environment for an extended duration, facilitating a gentle and phased release. Furthermore, the slow swelling induces a more temperate change in steric hindrance, necessitating a longer period for SPTH to overcome this barrier for release, which supports the phased release of SPTH [20,21].

### 2.5. Characterization of SPTH-SPC Microcapsules

#### 2.5.1. SEM of SPTH-SPC Microcapsules

**Figure 2:**
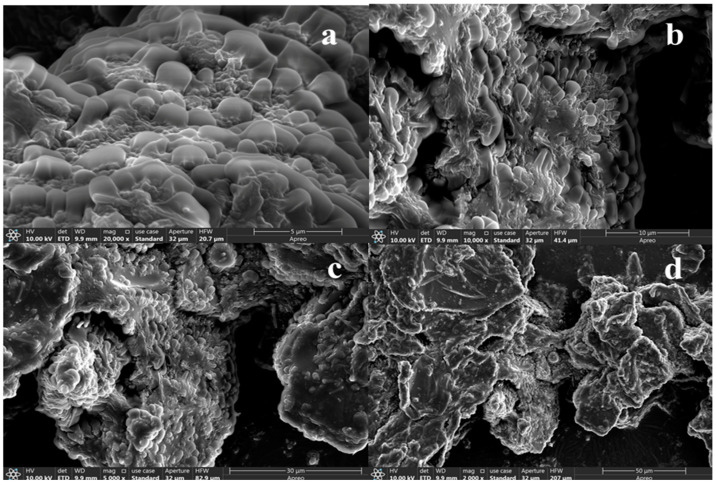
SEM of SPTH-SPC microcapsules at different magnifications. (**a**) 20,000×; (**b**) 10,000×; (**c**) 5000×; (**d**) 2000×.

The surface morphology of SPTH-SPC microcapsules was observed using SEM (Figure 2). The microcapsules displayed an uneven and spherical shape, characterized by an inwardly concave and aggregated surface. The SPTH-SPC microcapsules exhibited a block-like shape with a rough surface. A spherical structure interspersed with vacuoles of varying sizes was clearly visible on the blocky surface. SPTH was successfully encapsulated within the hydrophobic cavities provided by SPC to form these microcapsules, aligning with the findings of Cian et al. [22].

#### 2.5.2. FTIR of SPTH-SPC Microcapsules

**Figure 3:**
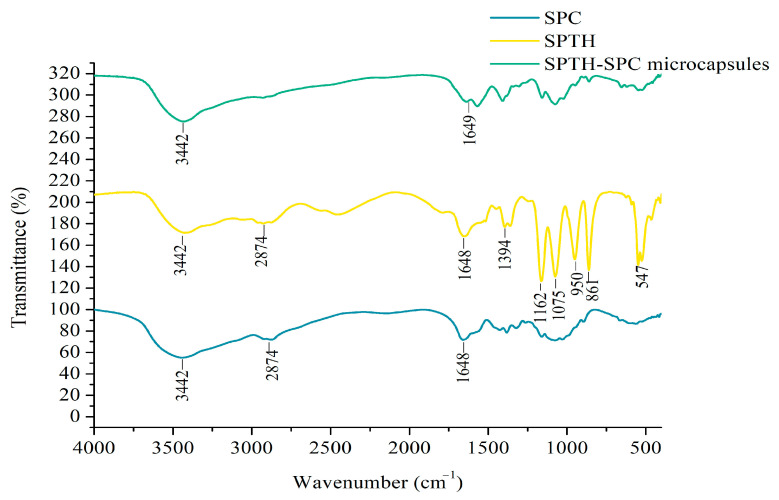
FTIR of SPC, SPTH, and SPTH-SPC microcapsules.

The SPTH-SPC microcapsules were characterized using FTIR and compared with SPC and SPTH. The results were presented in Figure 3. The FTIR analysis of SPC exhibited typical absorption peaks at 3442 and 2874 cm^−1^, corresponding to the stretching vibrations of O-H and C-H, respectively. The presence of -NH_3_^+^ was evidenced by the peak at 1560 cm^−1^, confirming the presence of β-chitosan. Additionally, absorption peaks at 1150 and 1080 cm^−1^ were observed, attributed to the symmetric deformation vibration of C-O-C and the stretching vibration of C-O. In the FTIR spectrum of SPTH, peaks at 1648 and 1394 cm^−1^ indicated the C=O stretching vibration of free carboxyl groups and the C-N stretching vibration (amide III band), respectively. The FTIR spectrum of SPTH-SPC microcapsules revealed the absence of the -NH_3_^+^ peak, providing evidence of electrostatic interaction between SPC and SPTH, resulting in the aggregation of SPTH-SPC microcapsules [23].

Notably, the FTIR spectrum of SPTH-SPC microcapsules showed a disappearance of most characteristic absorption peaks of SPTH, suggesting that the stretching vibration of the SPTH molecule was restricted after complexing with SPC, indicating encapsulation of SPTH in SPC. Peaks at 1075, 950, and 861 cm^−1^ in the FTIR spectra of SPTH disappeared, while a new peak emerged at 1649 cm^−1^ in the FTIR spectra of SPTH-SPC microcapsules. This finding aligns with a previous study that observed similar changes in the FTIR spectra of quinoa starch and rutin [24]. Furthermore, the disappearance of peaks at 1162, 1075, 950, and 861 cm^−1^ in the FTIR spectra of SPTH upon complexation with SPC suggested interactions between SPTH and SPC through phenolic OH groups during encapsulation.

The DD for the conversion of β-chitin to β-chitosan was determined to be 82.14%.

#### 2.5.3. XRD of SPTH-SPC Microcapsules

**Figure 4:**
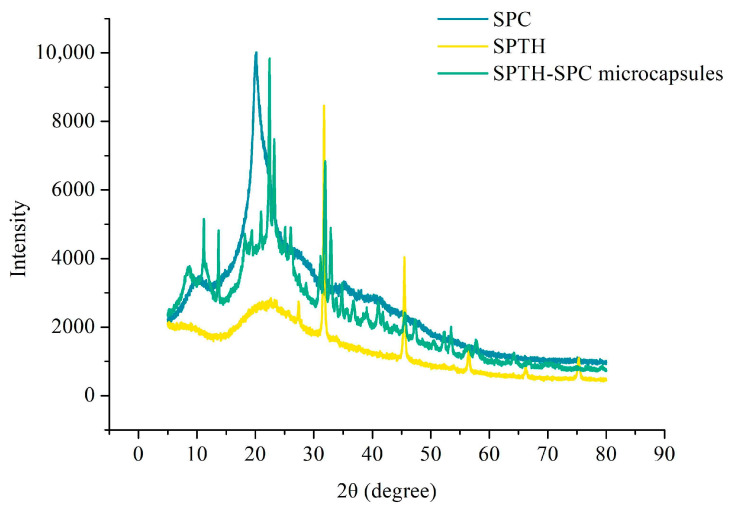
XRD of SPC, SPTH, and SPTH-SPC microcapsules.

XRD is a common technique used to analyze the crystallinity of powders. Amorphous samples typically display broad diffused peaks in their XRD pattern, whereas crystalline materials exhibit sharp peaks [25]. The relative crystallinity of individual components and microcapsule powders was evaluated using XRD, as presented in Figure 4. The core material SPTH exhibited a broad peak at around 20°, which was indicative of non-crystalline structures. In contrast, the wall material SPC exhibited multiple diffraction peaks in the range of 10–40°, suggesting the presence of a crystalline structure, consistent with previous studies on nanoparticles synthesized from soy protein [26,27]. Interestingly, the XRD patterns of the microcapsules did not exhibit the sharp characteristic peak of SPC, instead, a broader peak around 20° indicated an amorphous form, confirming the encapsulation of SPTH within the wall material. These findings supported the formation of the microcapsule structure and were consistent with the experimental results obtained from FTIR analysis.

#### 2.5.4. DSC of SPTH-SPC Microcapsules

**Figure 5:**
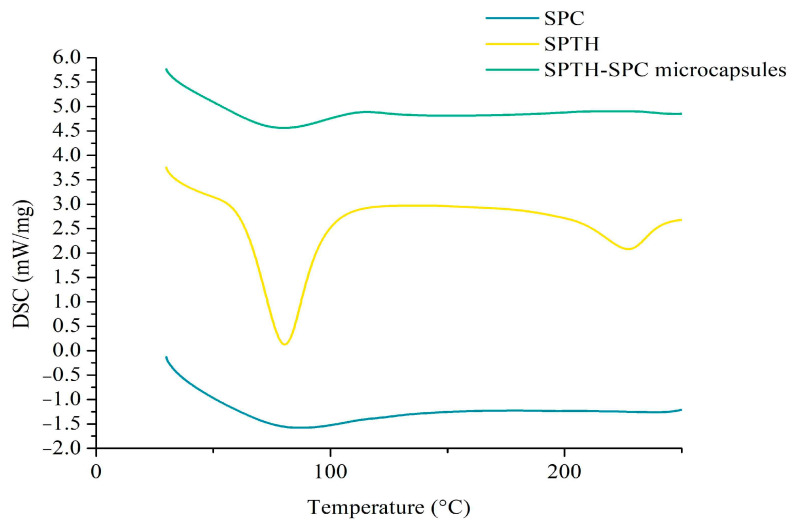
DSC of SPC, SPTH, and SPTH-SPC microcapsules.

DSC is a method used to analyze the thermal stability and degradation process of products within a specific temperature range [28]. Figure 5 depicted the DSC curves of SPTH-SPC microcapsules along with their wall and core materials. The maximum absorption peaks of SPC, SPTH, and SPTH-SPC microcapsules appeared at 87 °C, 80 °C, and 86 °C, respectively. The shift in peak positions suggested the successful loading of SPTH onto SPC. The higher thermal denaturation temperature of SPTH-SPC indicated that the microcapsules exhibited improved thermal stability compared to those prepared solely with protein. This enhancement is probably attributed to the structural reorganization of SPC and SPTH after complex formation, as well as the electrostatic attraction and hydrogen bonding between SPC and SPTH. These findings were consistent with results from a previous study [29].

#### 2.5.5. Particle Size and ζ-Potential of SPTH-SPC Microcapsules

**Figure 6:**
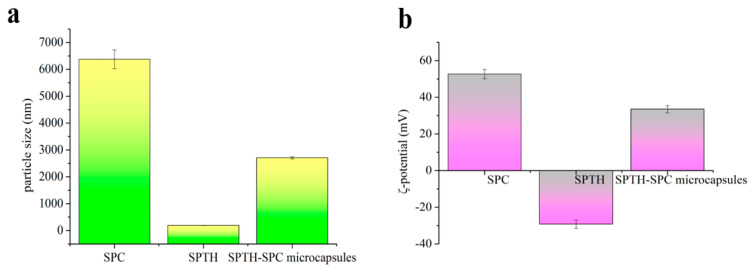
Particle size and ζ-potential of SPC, SPTH, and SPTH-SPC microcapsules: (**a**) Particle size; (**b**) ζ-potential.

The particle size distribution pattern of microcapsules is a critical parameter for their applications [30]. Figure 6a illustrates the impact of different ingredients on the particle size distribution pattern of microcapsules. The particle size distribution of SPTH was narrow, ranging from 185 to 195 nm with an average particle size of 190.12 nm. On the other hand, SPC exhibited a wider distribution with sizes between 6126 and 6625 nm and an average size of approximately 6375.89 nm. The microcapsules prepared with both ingredients had sizes ranging from 2679 to 2739 nm, indicating that the ultrasonic emulsification process led to a reduction in the particle dispersion of chitosan, resulting in SPTH being completely encapsulated in SPC. Figure 6b shows the ζ-potential of SPC, SPTH, and SPTH-SPC microcapsules. The ζ-potential reflects the positive or negative charges present on the particle surface, with higher absolute values indicating greater stability and resistance to aggregation [31]. The ζ-potential of SPC-SPTH exceeded 30 mV, indicating excellent stability of the SPTH-SPC microcapsules.

### 2.6. Antioxidant Activity In Vitro of SPTH-SPC Microcapsules

**Figure 7:**
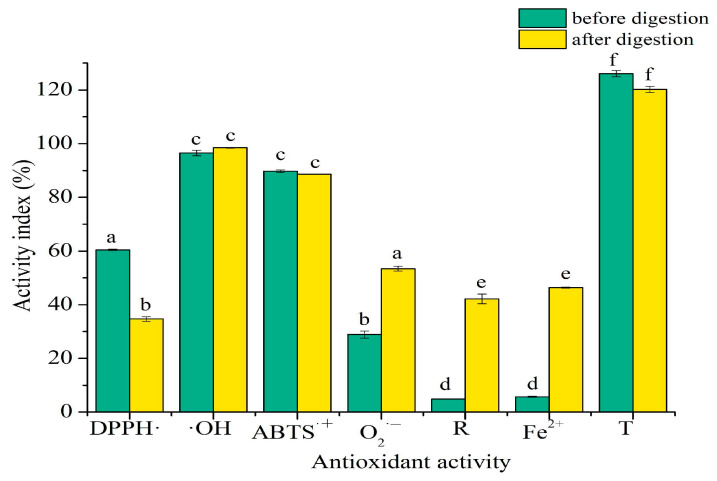
Antioxidant activity of SPTH-SPC microcapsules before and after digestion. DPPH· represents DPPH· radical scavenging activity, ·OH represents hydroxyl radical scavenging activity, ABTS^·+^ represents ABTS^·+^ scavenging activity, O_2_^·−^ represents O_2_^·−^ scavenging activity, R represents reducing power, Fe^2+^ represents chelating activity of ferrous ions, T represents total antioxidant activity. The lowercase letters represent significant differences (*p* < 0.05) in the activity of SPC-SPTH microcapsules before and after simulated gastric and intestinal digestion in vitro.

The protective effect of microcapsules on the antioxidant activities of SPTH during digestion was investigated by analyzing the antioxidant activity of simulated gastric and intestinal digestion of free SPTH [32,33,34]. Previous research demonstrated that after simulated digestion, the DPPH radical scavenging activity of unwrapped SPTH dropped to 0 [4]. As depicted in Figure 7, despite a decrease in DPPH radical scavenging activity post digestion, it still retained over 30%. This suggests that SPTH was encapsulated to enhance DPPH radical scavenging activity. Furthermore, the hydroxyl radical scavenging activity and ABTS^·+^ scavenging activity remained stable. The study also revealed that under both gastric and intestinal simulated conditions, the O_2_^·−^ scavenging activity, reducing power, chelating activity of ferrous ions, and total antioxidant activity of the digested SPC-SPTH microcapsules were significantly (*p* < 0.05) higher than those of undigested microcapsules. The superior antioxidant activity of the SPTH-SPC microcapsules compared to undigested microcapsules may be attributed to the slow-release effect of the microcapsules during digestion [35]. These findings indicated that the encapsulation provided good protection for the bioactivity of SPTH. After simulated digestion of gastroenteric fluid, the antioxidant activity of the microcapsules was significantly enhanced, showing a significant difference from that of undigested microcapsules, which was consistent with the cumulative release of SPTH in gastric and intestinal simulated juices [23]. Similar findings have been reported with chitosan-encapsulated polyphenols, where the polymer matrix delayed the exposure of core materials to harsh conditions, thereby preserving their bioactivity [36,37]. Notably, the observed increase in O_2_^·−^ scavenging activity post-digestion may result from the gradual breakdown of the SPC wall, releasing SPTH fractions with higher solubility and reactivity. This phenomenon aligns with studies on lipid-based vitamin E carriers, where nanoencapsulation enhanced radical scavenging efficiency by improving dispersibility [38]. Future research should focus on in vivo validation to assess whether this in vitro stability translates to physiological antioxidant effects.

## 3. Materials and Methods

### 3.1. Materials and Reagents

The Peruvian SP used in this study was acquired from Jinglu Fishery Co., Ltd. (Penglai, China) and transported to our laboratory under cold chain conditions. Prior to utilization, SP was washed with distilled water, dried in an oven at 30 °C, crushed into a powder, and stored in a desiccator. Trypsin (250,000 U/g) was obtained from Solarbio Biotechnology Co., Ltd. (Beijing, China). All other reagents and chemicals utilized in this study were of analytical grade and purchased from Sinopharm Chemical Reagent Co., Ltd. (Shanghai, China).

### 3.2. Determination of the Composition of SP

All the composition of SP was determined by analyzing moisture, protein, ash, and chitin content using various methods including direct drying, Kjeldahl nitrogen determination, high-temperature burning, and the Folin-phenol reagent method [39]. The amino acid composition was analyzed using an amino acid automatic analyzer after acid hydrolysis [40].

### 3.3. Preparation of SPTH and SPC

SP was hydrolyzed with trypsin at pH 5.5, a solid–liquid ratio of 1:10, a temperature of 40 °C, a duration of 6 h, and a protease addition of 11,000 U/g protein. The reaction was stopped by inactivating the sample at 100 °C for 10 min post hydrolysis. Following centrifugation at 10,000 rpm for 10 min, the supernatant was freeze-dried to obtain SPTH, while the precipitation was dried in an oven to obtain SP β-chitin [41]. SPC was generated through thermochemical alkaline deacetylation of β-chitin [42]. Specifically, 30 g of SP β-chitin was mixed with 300 mL of 40% NaOH solution and incubated for 3 h in a water bath at 80 °C. The resulting SPC was washed repeatedly until neutral and subsequently dried in an oven at 30 °C for 48 h. The degree of deacetylation of the resulting β-chitosan was calculated to be 84.12%, based on the FTIR results.

### 3.4. Preparation of Microcapsules

SPTH-SPC microcapsules were prepared using a physical-mechanical method as described in the literature with some modifications [7]. A specific quantity of SPC was dispersed into a 1% acetic acid solution, heated, and stirred until fully dissolved. Subsequently, a specific quantity of SPTH was gradually added and thoroughly mixed. The solution underwent ultrasonic treatment for a defined duration before being freeze-dried at −65 °C for 48 h to obtain SPTH-SPC microcapsule powder. The study focused on investigating encapsulation efficiency based on four independent variables: SPC concentration (1%, 1.5%, 2%, 2.5%, 3%), SPTH concentration (2, 4, 6, 8, 10 mg/mL), core-to-wall ratio (1:2, 1:4, 1:6, 1:8, 1:10), and sonication time (10, 20, 30, 40, 50 min). The experimental was designed as a single-factor experiment, followed by a four-factor, three-level orthogonal experimental design utilizing the Orthogonal Design Assistant V3.1 software.

### 3.5. Determination of Encapsulation Efficacy

A total of 0.2 g of microcapsules were rinsed with distilled water and then centrifuged at 5000 rpm for 10 min. The resulting precipitate was dissolved in 0.5% acetic acid, and the SPTH content was determined using the Lowry method [43]. Absorption values were plotted on a standard curve (Y = 0.3405X − 0.0109, R^2^ = 0.9991) to calculate the SPTH content. The standard curve was generated by preparing a 2 mg/mL bovine serum protein standard solution and measuring absorption values at different protein concentrations. Encapsulation efficacy was calculated using the formula:

(1) $$Encapsulation\;efficacy\;(\%)=\frac{p_{1}}{p_{2}}\times 100$$

where *p*_1_ represents the amount of SPTH embedded in microcapsules and *p*_2_ represents the amount of SPTH added to the microcapsules.

### 3.6. Determination of Dispersibility

The microcapsules, weighing 1 g, were allowed to flow freely through a funnel onto a horizontal disk, forming a conical pile. The radius and height of the pile were subsequently measured to calculate the angle of repose (ϕ) of the microcapsules. The angle of repose (ϕ) was calculated using the formula:

(2) $$\phi =\arctan\frac{h}{r}$$

where *h* represents the height of the conical powder stack, mm; and *r* represents the radius of the conical powder stack, mm.

### 3.7. Determination of Stacking Density

The prepared microcapsule samples were placed in a 10 mL measuring cylinder and shaken horizontally to allow the microcapsule powder to settle naturally. The mass and volume of the filled microcapsules were recorded to determine the stacking density. Stacking density was calculated as the mass of microcapsules per unit volume using the formula:

(3) $$d=\frac{m}{V}$$

where *d* represents the stacking density of microcapsules, g/mL; *m* represents the mass of microcapsules, g; and *V* represents the volume of the filled microcapsules, mL.

### 3.8. In Vitro Release Patterns During Simulated Gastrointestinal Digestion

The previous method involved quantifying the total SPTH released from SPTH-SPC microcapsules [44]. To simulate the artificial gastric environment, pepsin was dissolved in deionized water (1 mg/mL) and the pH was adjusted to 2.0 using 1 mol/L HCl. For the artificial intestinal environment, trypsin was dissolved in deionized water (2 mg/mL) and the pH was adjusted to 7.4 using 1 mol/L NaOH. In this experiment, 100 mg microcapsules were added to 100 mL of a simulated gastric solution. The mixture was incubated at 37 °C for 2 h. Every 30 min, 1 mL of artificial gastric fluid was sampled and replaced with 1 mL of fresh artificial gastric fluid to determine the amount of SPTH released. After 2 h, the pH of the artificial gastric juice was adjusted to 7.4 with 1 mol/L NaOH before introducing the simulated intestinal fluid. The simulated intestinal digestion process was carried out for another 2 h. The release behavior of the microcapsules in the simulated gastric and intestinal fluids was calculated using the following equations:

(4) $$\mathrm{Release}\;\mathrm{ratio}\;\mathrm{in}\;\mathrm{stimulated}\;\mathrm{gastric}\;\mathrm{fluids}\;(\%)=\frac{m_{1}}{m_{2}\times \;\mathrm{encapsulation}\;\mathrm{efficacy}\;(\%)}\times 100$$

(5) $$\mathrm{Release}\;\mathrm{ratio}\;\mathrm{in}\;\mathrm{stimulated}\;\mathrm{intestinal}\;\mathrm{fluids}\;(\%)=\frac{m_{3}}{m_{4}\times \;\mathrm{encapsulation}\;\mathrm{efficacy}\;(\%)}\times 100$$

where *m*_1_ represents the release amount of SPTH in simulated gastric fluids, g; *m*_2_ represents the mass of microcapsules, g; *m*_3_ represents the release amount of SPTH in simulated intestinal fluids, g; and *m*_4_ represents the mass of microcapsules, g. The mass was determined and calculated using the Folin-phenol-ninhydrin method [4].

### 3.9. Characterization of SPTH-SPC Microcapsules

#### 3.9.1. Fourier Transform Infrared Spectroscopy (FTIR) and the Degree of Deacetylation (DD)

The FTIR spectra of SPTH, SPC, and SPTH-SPC microcapsules were recorded using KBr pellets on a NICOLET-380 infrared spectrometer (Thermo Fisher, Waltham, MA, USA) in the wavenumber range of 4000–400 cm^−1^ [45].

The amide III band was selected as the analytical band, with the reference band set at 1420 cm^−1^ for a more accurate determination of the DD of the sample. This method helps eliminate interference from water, residual acid, or residual alkali in the sample. The DD calculation was based on the formula:

(6) $$\mathrm{DD}\left(\%\right)=\left(1.122-0.3192\times \frac{A_{1320}}{A_{1420}}\right)\times 100$$

where *A*_1320_ represents the absorbance of FTIR at 1320 cm^−1^ and *A*_1420_ represents the absorbance of FTIR at 1420 cm^−1^.

#### 3.9.2. Scanning Electron Microscopy (SEM)

The microstructural characteristics of SPTH-SPC microcapsule powder were examined using a Zeiss Sigma 300 ultra-high resolution scanning electron microscope (Carl Zeiss, Oberkochen, Germany) [46]. Prior to electron microscope scanning, the samples were sprayed with gold and mounted on a short aluminum rod to capture micrographs of the samples.

#### 3.9.3. X-Ray Diffraction (XRD)

XRD analysis was conducted using a D8 ADVANCE instrument (Bruker, Billerica, MA, USA) with Cu-Kα radiation as the excitation source. A total of 2 g of microcapsules were compressed into pellets for the analysis, with a scanning range from 5° to 80° (2θ) at a rate of 5°/min [13].

#### 3.9.4. Differential Scanning Calorimeter (DSC)

For DSC analysis, approximately 10 mg of SPTH-SPC microcapsules were placed in a sealed aluminum crucible and analyzed using a PerkinElmer DSC8000 instrument (PerkinElmer, Taicang, China) with a heating rate of 10 °C/min, temperature range of 20–200 °C, and nitrogen gas flow rate of 20 mL/min [47].

#### 3.9.5. Particle Size and Zeta Potential of the SPTH-SPC Microcapsules

Particle size and zeta potential of the nanoparticles were measured using a Zetasizer Nano-ZS (Nono Brook, 90 Plus PALS, Holtsville, NY, USA) based on DLS techniques [48]. All measurements were performed in triplicate at a temperature of 25 ± 1 °C.

### 3.10. Antioxidant Activity Assay In Vitro

#### 3.10.1. DPPH Radical Scavenging Activity Assay

The DPPH radical scavenging activity of the microcapsules was assessed following the methodology outlined by Ge et al. [49], with minor modifications. Specifically, 2 mL of a 20 mg/mL sample solution was mixed with 2 mL of the 0.2 mM DPPH anhydrous ethanol solution, and the absorption value was determined at a wavelength of 517 nm after 30 min of reaction at 25 °C. The DPPH radical scavenging activity was then calculated using the formula:

(7) $$\mathrm{DPPH}\;\mathrm{radical}\;\mathrm{scavenging}\;\mathrm{activity}\;\left(\%\right)=(1-\frac{A_{1}-A_{2}}{A_{0}})\times 100$$

where *A*_0_ is the absorbance of anhydrous ethanol without the sample, *A*_1_ is the absorbance of the sample, and *A*_2_ is the absorbance of anhydrous ethanol without DPPH.

#### 3.10.2. Hydroxyl Radical Scavenging Activity Assay

The hydroxyl radical scavenging activity was assessed following a modified protocol based on Qing et al. [50]. Specifically, 1.0 mL of the sample was combined with 1.0 mL of FeSO_4_ solution (2 mM), 1.0 mL of a salicylic acid-ethanol solution (6 mM), and 1 mL of 6 mM H_2_O_2_ solution. The mixture was thoroughly mixed and then incubated at 37 °C for 30 min. Subsequently, the absorption value was measured at 510 nm. The hydroxyl radical scavenging activity was calculated using the formula:

(8) $$\mathrm{Hydroxyl}\;\mathrm{radical}\;\mathrm{scavenging}\;\mathrm{activity}\;\left(\%\right)=(1-\frac{A_{1}-A_{2}}{A_{0}})\times 100$$

where *A*_0_ is the absorbance of distilled water in place of the sample, *A*_1_ is the absorbance of distilled water in place of the hydrogen peroxide solution, and *A*_2_ is the absorbance of the sample.

#### 3.10.3. ABTS^·+^ Scavenging Activity Assay

The ABTS^·+^ scavenging activity was determined following the method described by Wang et al. [51]. A mixture of ABTS solution (1 mL, 7 mM) and K_2_S_2_O_8_ (1 mL, 2.45 mM) was prepared and incubated at 4 °C for 12 h in the dark to generate a blue-green ABTS^·+^ solution. This solution was then diluted with 95% ethanol to achieve a background absorbance of 0.70 ± 0.05. The sample was dissolved in deionized water (80 μL) and mixed with the blue-green ABTS^·+^ solution. The resulting mixture was incubated at 37 °C for 10 min in the dark, and the absorbance was measured at 734 nm. The ABTS^+^ scavenging activity of the microcapsules was calculated using the formula:

(9) $${\mathrm{ABTS}}^{\cdot +}\mathrm{scavenging}\;\mathrm{activity}\left(\%\right)=(1-\frac{A_{1}}{A_{0}})\times 100$$

where *A*_0_ is the absorbance of the blank group and *A*_1_ is the absorbance of the sample group.

#### 3.10.4. O_2_^−^ Scavenging Activity Assay

The O_2_^−^ scavenging activity was assessed following the method outlined by Zhou et al. [52], with minor modifications. Specifically, 0.5 mL of the sample solution was mixed with 5.0 mL of Tris-HCl buffer (50 mM, pH 8.2) and incubated at 25 °C. Subsequently, 0.5 mL of 3 mM pyrogallic acid was introduced to the mixture, and absorbance values were measured at 325 nm. The O_2_^−^ scavenging activity was calculated using the formula:

(10) $${O_{2}}^{-}\;\mathrm{scavenging}\;\mathrm{activity}\;\left(\%\right)=(1-\frac{A_{1}}{A_{0}})\times 100$$

where *A*_0_ is the absorbance of the blank group and *A*_1_ is the absorbance of the sample group.

#### 3.10.5. Reducing Power Assay

The reducing power was determined using a previously established method [53,54]. In this method, 1 mL of sample was combined with 2.5 mL of potassium ferricyanide (1%), 500 μL of phosphate buffer solution (0.2 M, pH 6.6), and then incubated at 50 °C for 20 min. The mixture was then cooled to room temperature and 5 mL of a 10% trichloroacetic acid solution was added. After centrifugation at 3000 rpm for 10 min, the supernatant (100 μL) was mixed with deionized water (250 μL) and ferric chloride solution (50 μL, 0.1%). Following a 10 min incubation period, the absorbance was measured at 700 nm. The reducing power was calculated using the formula:

(11) $$\mathrm{Reducing}\;\mathrm{power}\;\left(\%\right)=(1-\frac{A_{1}}{A_{0}})\times 100$$

where *A*_0_ is the absorbance of the blank group and *A*_1_ is the absorbance of the sample group.

#### 3.10.6. Ferrous Ions Chelating Activity Assay

The chelating activity of ferrous ions was evaluated using a method described in previous studies [55]. A sample solution (100 μL), 2 mM FeCl_2_ (5 μL), and distilled water (135 μL) were mixed in a tube. The mixture was then incubated at room temperature in the dark for 3 min, followed by the addition of a 5 mM phenelzine solution (10 μL). After thorough blending, the absorbance was measured at 562 nm after 10 min. The chelating activity of ferrous ions was calculated using the formula:

(12) $$\mathrm{Ferrous}\;\mathrm{ions}\;\mathrm{chelating}\;\mathrm{activity}\;\left(\%\right)=(1-\frac{A_{1}}{A_{0}})\times 100$$

where *A*_0_ is the absorbance of the blank group and *A*_1_ is the absorbance of the sample group.

#### 3.10.7. Total Antioxidant Activity Assay

Total antioxidant activity was assessed using the FRAP method, following the protocol outlined by Chen et al. [54]. A 1 mL sample was mixed with 3 mL of the FRAP reagent and left to react in darkness at room temperature. The absorbance at 593 nm was recorded after 30 min of reaction.

### 3.11. Statistic Analysis

Each experiment was repeated a minimum of three times. Variations between samples were statistically analyzed using ANOVA, with significance set at *p* < 0.05.

## 4. Conclusions

SPTH-SPC microcapsules were prepared using SPC as encapsulating materials through ultrasonic emulsification. Analysis through FTIR, XRD, DSC, and SEM demonstrated the successful encapsulation of SPTH within the SPC complex, resulting in the formation of SPC-SPTH microcapsules characterized by small particle size, good thermal stability, and high encapsulation rate. These microcapsules protected SPTH from gastric pepsin digestion in the stomach while enabling its release in the intestine, thereby improving its bioavailability. The development of SPTH-SPC microcapsules presents a novel application for SPTH in various fields, laying the groundwork for hydrophobic compounds like SPTH with high bioactive effects but limited bioavailability and sensitivity to gastric conditions. Further research is necessary to explore in vivo biological activity.

## Figures and Tables

**Table 1:** SP composition.

Component	Content (%)
Water	6.02 ± 0.76
Ash	3.26 ± 0.22
Protein	64.41 ± 1.24
Chitin	26.03 ± 0.73

**Table 2:** Amino acid composition of SP.

Amino Acid Separation	Amino Acid Name	Content (g/100 g)
Essential amino acid (EAA)	Val	3.48
	Ile	1.77
	Leu	4.2
	Phe	1.06
	Met	13.67
	Thr	2.18
	Lys	2.18
Non-essential amino acid (NEAA)	Glu	2.67
	Asp	4.14
	Ala	6.41
	Gly	5.82
	Tyr	9.81
	His	7.12
	Ser	1.89
	Arg	1.18
	Cys	1.74
	Pro	6.68
EAA		28.53
NEAA		47.46
Total amino acids (TAA)		75.99
EAA/TAA (%)		37.55
EAA/NEAA (%)		60.13

**Table 3:** Optimal preparation conditions for SPTH-SPC microcapsules and their encapsulation efficiency.

SPC Concentration	SPTH Concentration	Core-to-Wall Ratio	Sonication Time	Encapsulation Efficiency
2%	4 mg/mL	1:3	20 min	58.95 ± 2.53%

## Data Availability

The datasets generated and analyzed during the current study are available from the corresponding authors upon reasonable request.
